# A Reference Matching-Based Temperature Compensation Method for Ultrasonic Guided Wave Signals

**DOI:** 10.3390/s19235174

**Published:** 2019-11-26

**Authors:** Geng Wang, Yuhang Wang, Hu Sun, Bingrong Miao, Yishou Wang

**Affiliations:** 1 School of Aerospace Engineering, Xiamen University, Xiamen 361005, China; wg.g@outlook.com (G.W.); peimys@163.com (Y.W.); sunhu@xmu.edu.cn (H.S.); 2 College of Mechanical and Automotive Engineering, Zhaoqing University, Zhaoqing 526061, China; 3 State-key Laboratory of Traction Power, Southwest Jiaotong University, Chengdu 610031, China; brmiao@home.swjtu.edu.cn

**Keywords:** structural health monitoring, ultrasonic guided wave, temperature compensation, reference signal matching, composite material

## Abstract

The ultrasonic guided wave-based structural damage diagnosis method has broad application prospects in different fields. However, some environmental factors such as temperature and loads will significantly affect the monitoring results. In this paper, a reference matching-based temperature compensation for ultrasonic guided wave signals is proposed to eliminate the effect of temperature. Firstly, the guided wave signals measured at different temperatures are used as reference signals to establish the relationship between the features of the reference signals and temperature. Then the matching algorithm based on Gabor function is used to establish the relationship between the amplitude influence coefficient obtained by the reference signal and the corresponding temperature. Finally, through these two relationships, the values of the phase and amplitude influence coefficients of the guided wave signals at other temperatures are obtained in a way of interpolation in order to reconstruct the compensation signals at the temperature. The effect of temperature on the amplitude and phase of the guided wave signal is eliminated. The proposed temperature compensation method is featured such that the compensation performance can be improved by multiple iteration compensation of the residual signal. The ultrasonic guided wave test results at different temperatures show that the first iterative compensation of the proposed method can achieve compensation within the temperature range greater than 7 °C, and the compensation within the temperature range greater than 18 °C can be achieved after three iterations.

## 1. Introduction

Ultrasonic guided waves have the characteristics of long propagation distance, small attenuation, large detection range, and are sensitive to damage such as cracks, debonding, and delamination. Ultrasonic guided waves have broad application prospects in damage monitoring of large-area metal and composite structures such as wings and fuselages [1,2,3,4,5,6,7,8,9].

However, ultrasonic guided waves are susceptible to environmental factors such as temperature and stress during the propagation process, which may lead to false reports and false positives. Therefore, it is an important challenge of ultrasonic guided wave structure health monitoring technology to eliminate or compensate for the influence of environmental factors on the ultrasonic guided wave diagnosis results [10,11,12]. Studies have shown that temperature is an important factor affecting the propagation characteristics of ultrasonic guided waves [13,14,15,16].

Many scholars have carried out a lot of research on the temperature compensation of ultrasonic guided waves from the perspective of experiment and theoretical analysis, and developed various temperature compensation methods, Lu and Michaels [17] proposed the baseline signal stretch (BSS) method in 2005 for guided wave ambient temperature compensation. The method can achieve a 95% damage detection rate when the temperature changes more than 30 °C, but the signal frequency change will result in poor compensation effect, and the compensation performance is highly dependent on the modal purity and signal complexity, and the maximum temperature interval for compensation is 5 °C.

In 2007, Konstantinidis et al. [18] proposed the optimal baseline subtraction (OBS) method, which allows the environment to change greatly. Compared with BSS, the residual signal level can be reduced by 10 dB, which significantly improves the long-term stability of the structural health monitoring (SHM) system. However, in order to ensure good damage sensitivity, the reference signal temperature interval is small (0.1 °C), making a large amount of reference data.

Lu et al. [19] (2010) optimized the OBS method to effectively compensate for temperature effects in short time windows and low sampling rates, and the damage detection performance was also significantly improved. Harley et al. [20] (2012) developed a new model drive and scale transformation based on OBS method, which can significantly improve computational efficiency and can be used for temperature compensation at sensor arrays and compensation of non-uniform temperature fields. Wang et al. [21] (2014) proposed an ultrasonic guided wave temperature compensation method based on adaptive digital filtering and OBS, which can effectively realize temperature compensation in a large temperature change interval (at least 10 °C) and has good effectiveness and robustness.

Although the methods on the basis of reference signal transformation can avoid the direct analysis of the original signals, they can compensate the temperature influence to a certain extent. However, it is difficult to achieve a large benchmark database that can cover the environmental impact of the normal structure. The methods based on the reference signal transformation cannot get rid of the constraints of signal complexity and modal purity, which limits the practical application of these methods.

Since 2011, Elizabeth et al. [22] used the cointegration method in econometrics to perform temperature compensation analysis, Dao and Staszewski [23] (2013) and Worden et al. [24] (2014) both compensated the effect of temperature by improved cointegration methods. These methods are simple for signal processing and do not lose feature information, but are only applied to linear correlation variables, and require pre-training data, and training data selection is also limited.

Yan et al. [25,26] (2005) successfully applied the PCA (Principal Component Analysis) method to compensate for the influence of temperature. This method does not need to measure environmental parameters, and it is simple and effective to deal with both linear and nonlinear systems, but the processing and calculation process of signals is complicated and very time consuming. In 2014, Kullaa et al. [27] adopted the Gaussian mixture model (GMM) to compensate for the nonlinear effects caused by the environment and can compensate for the nonlinear environmental impact well under low-dimensional data. However, the data training amount is large and the compensation performance under high-dimensional data is not as good as that of the PCA method.

In 2015, Roy et al. [28] established a relationship between the matching factor and the material characteristic parameters of the environmental impact based on the matching tracking algorithm proposed by Lu [29] (MP, Matching Pursuits ) and then reconstructed the signal under the influence of the environment use the relationship obtained from the training data set to achieve compensation. However, this method still did not get rid of the huge amount of data and computational complexity of the MP algorithm construction process. In addition, for non-uniform temperature environments, Sun et al. [30] (2019) proposed a compensation method for Lamb wave-based damage detection within a non-uniform temperature field. The Hilbert transform and Levenberg–Marquardt optimization algorithm are developed to extract the amplitude and phase variation caused by the change of temperature, which is used to establish a data-driven model for reconstructing the reference signal at a certain temperature.

Studies have shown that the effect of temperature on guided wave signals is reflected in the change of their amplitude and phase (i.e., the time of arrival) [31,32], that is, the temperature increasing will cause the amplitude of the guided wave signal to decrease and the time of arrival (corresponding phase) to increase. In this paper, a temperature compensation method based on reference signal matching is proposed for guided wave signals. Firstly, the guided wave signals measured at equal temperature intervals constitute a set of reference signals, where a signal in the set is selected as the only atom in the Gabor function dictionary, and then matched with other reference signals to obtain a relationship between the influence coefficient of the signal amplitude and the temperature. Then, the amplitude influence coefficient at other temperatures outside the reference temperature range is calculated by interpolation, so as to compensate for the influence of temperature on the amplitude of the guided wave signal, and the compensation of the entire guided wave signal is realized on this basis. The method is characterized by simple calculation, and the temperature compensation can be realized by using a small number of data sets, the compensation temperature range is large, and the compensation cost is low.

## 2. Theoretical Background

**Figure 1:**
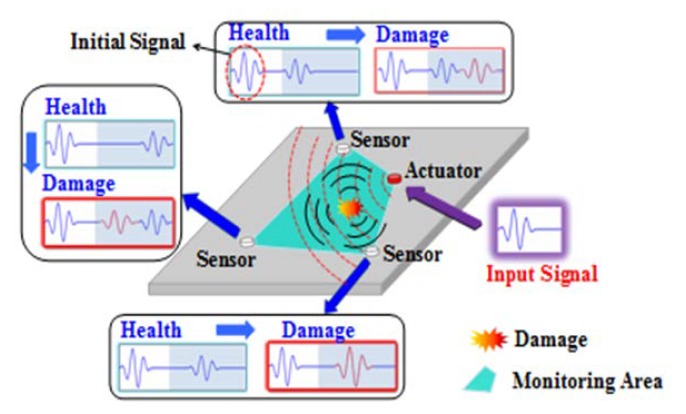
Schematic diagram of ultrasonic guided wave-based structural health monitoring (GWSHM) damage diagnosis principle.

The basic principle of ultrasonic guided wave-based structural health monitoring (GWSHM) to realize damage monitoring in the plate structure is to use the sensor network pre-arranged on the structure to generate guided waves on the surface of the structure to be inspected by the excitation end. If there is damage on the surface or inside the structure, the propagation characteristics of the guided wave will change. Then, the guided wave signal obtained at the receiving end is compared with the reference signal (generally the signal in the structural health state), and the damage diagnosis of the structure can be realized by analyzing the changes of these features. Figure 1 shows the schematic diagram of the GWSHM implementation of damage diagnosis.

However, the changes of the signals are not always caused by damage, it may also be caused by environmental factors such as temperature and load. Temperature affects the test piece, the sensor, and the glue layer between them, causing changes in signal characteristics. These changes are mixed with the changes caused by the damage and are ultimately reflected in the changes in signal propagation characteristics. The compensation method proposed in this paper is to use the relationship between the characteristic change of the signal and the temperature. This compensation method is for the signal itself, and the effects of temperature on the sensor and the test piece, etc., are already reflected in the signal and are taken into account.

## 3. Reference Matching-Based Compensation Method

The proposed method is mainly based on the matching pursuit algorithm of Gabor function. Any guided signal, *S*, can be expressed as in Equation (1):

(1) $$S=\sum_{k=0}^{n-1}\langle R^{k}S,g_{\gamma_{k}}\rangle g_{\gamma_{k}}+R^{n}S,$$

where n is the number of iterations, $g_{\gamma_{k}}$ is the Gabor atom selected for k iterations, $R^{n}S$ is the residual signal after *n* iterations, 〈.,.〉 is the vector inner product, and the value of $\left\|g_{\gamma_{k}}\right\|$ is always set as 1, where ‖.‖ represents the two norms of the vector.

Each Gabor atom is selected from the Gabor dictionary set, D, where D is an over-complete atomic library. The atom library is constructed by a Gaussian window function, as in Equation (2):

(2) $$g_{\gamma }\left(t\right)=\frac{1}{\sqrt{s}}g\left(\frac{t-u}{s}\right)\cos\left(vt+w\right),$$

where $g_{\gamma }\left(t\right)$ is a Gabor atom, $g\left(t\right)=e^{-\pi t^{2}}$ is a Gaussian window function, s and u are scale and translation factors, v is a frequency factor, and w is a phase factor, γ={s,u,v,w}.

In each process of approximating the signal s, it is necessary to ensure that the selected atom best matches the structure of the signal *s*, namely, Equation (3) should be satisfied to obtain an expected relationship.

(3) $$\left|\langle S,g_{\gamma_{0}}\rangle \right|=\sup\left|\langle s,g_{\gamma }\rangle \right|,$$

where the signal S is decomposed according to Equation (4):

(4) $$S=\langle S,g_{\gamma_{0}}\rangle g_{\gamma_{0}}+R^{1}S.$$

Generally speaking, the residual signal can be further decomposed according to Equation (5):

(5) $$R^{1}S=\langle R^{1}S,g_{\gamma_{1}}\rangle g_{\gamma_{1}}+R^{2}S.$$

Similarly, the decomposition process should also meet the following condition as in Equation (6):

(6) $$\left|\langle R^{1}S,g_{\gamma_{1}}\rangle \right|=\sup\left|\langle R^{1}S,g_{\gamma_{1}}\rangle \right|.$$

The iterative decomposition is performed by the above equations. The guided wave signal, S, can be expressed in the form expressed in Equation (1).

However, since complex guided wave signals are susceptible to environmental factors, directly analyzing the signal using Equation (1) can result in significant computational costs. Considering that the temperature changes will influence the phase and amplitude of signals, this paper uses the matching pursuit of different reference signals to characterize the signals at different temperatures. The proposed method will reduce the computational cost, that is, the guided wave signal, $S_{T},$ at any temperature can be expressed as the sum of reference signal, $S_{b},$ and residual signal, $RS_{T},$ as shown in Equation (7):

(7) $$S_{T}=S_{b}+RS_{T}.$$

If the reference signal is processed to satisfy the condition of the Gabor atom (i.e., $\|g_{\gamma }\|=1$), the reference signal atom, $g_{b},$ is obtained as expressed by Equation (8). Then the atom will contain all of the time–frequency characteristics of $S_{b}$ associated with the reference signal but differ in magnitude.

(8) $$g_{b}=\frac{S_{b}}{\left\|S_{b}\right\|},\|g_{b}\|=1$$

If the phase change is ignored, the difference between the reference signal and the signal at different temperatures is mainly reflected in the amplitude. Taking the reference atom, $g_{b},$ as the optimal matching atom under the current temperature signal expressed by Equation (4), the signal at any different temperature can be expressed as in Equation (9):

(9) $$S_{T}=\langle S_{T},g_{b}\rangle g_{b}+R^{1}S_{T},$$

where $S_{T}$ represents the guided wave signal measured at *T*(°C) and $R^{1}S_{T}$ represents the residual signal after the first compensation.

Let $S_{T},g_{b}$ be the coefficient related to the change of the amplitude of the guided wave signal. Then, the amplitude influence coefficient, Coef(T), can be defined as in Equation (10):

(10) $$Coef\left(T\right)=\langle S_{T},g_{b}\rangle .$$

The phase change (i.e., the time of arrival short for ToA) is directly determined by the feature point of the guided wave signal. In this study, the peak of the first arriving wave is selected as the feature point. Therefore, the functional relationship between the phase and the temperature change is established in Equation (11):

(11) ToA=f(T).

Through Equations (10) and (11), the values of Coef and ToA of the real signal at the non-reference temperature can be interpolated in order to compensate for the change of the amplitude and phase of the signal and eliminate the temperature influence.

**Figure 2:**
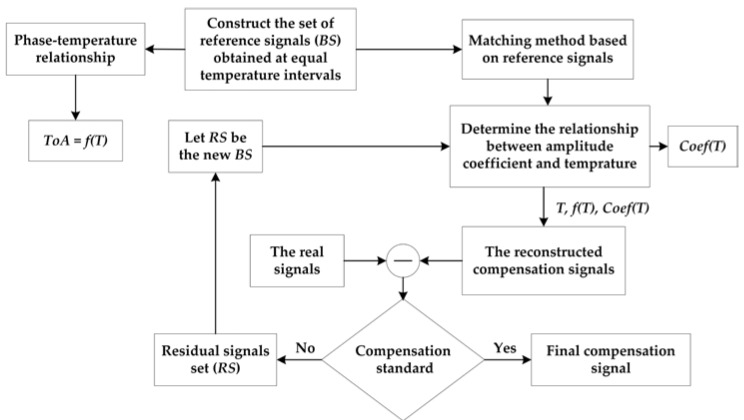
Flowchart of temperature compensation for ultrasonic guided wave signals.

If the first compensation signal does not meet the given compensation criteria, the second iteration compensation can be performed. The residual signal of the first compensated signal and the real signal is extracted according to Equation (5). The residual signal of the signal satisfying the compensation standard is used as a new reference signal set to establish an amplitude influence coefficient relationship and achieve compensation for the residual. This new compensation result is then superimposed on the first compensation signal to reconstruct the second literately compensated signal. If the compensation result still does not meet the given compensation criteria, repeat the above process. The compensation process of the proposed method is shown in Figure 2.

## 4. Results

### 4.1. Experiment Setup

**Figure 3:**
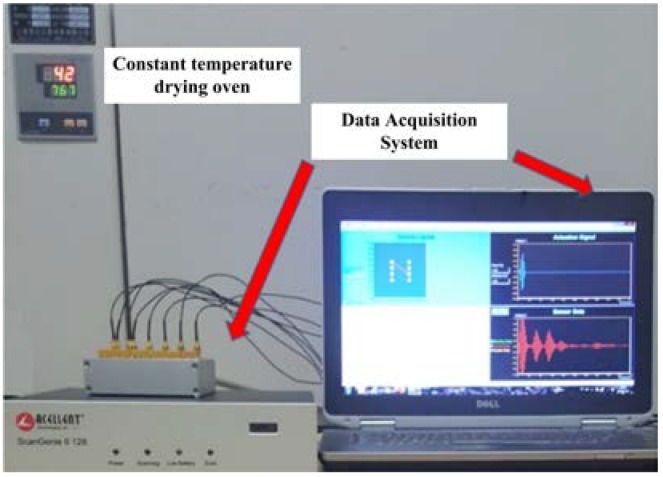
Environmental chamber and data acquisition system.

The experimental setup is shown in Figure 3. The data acquisition system is a 64-channel ScanGenie II developed by Acellent (Sunnyvale, CA, USA). The excitation guided wave is a five-cycle sinusoidal signal modulated by a Hanning window. In the whole data acquisition process, the sweep excitation signal is used, the signal center frequency varies from 100 to 240 kHz, the step size is 20 kHz, the sampling frequency is 24 MHz, and the sampling point is 12,000.

**Figure 4:**
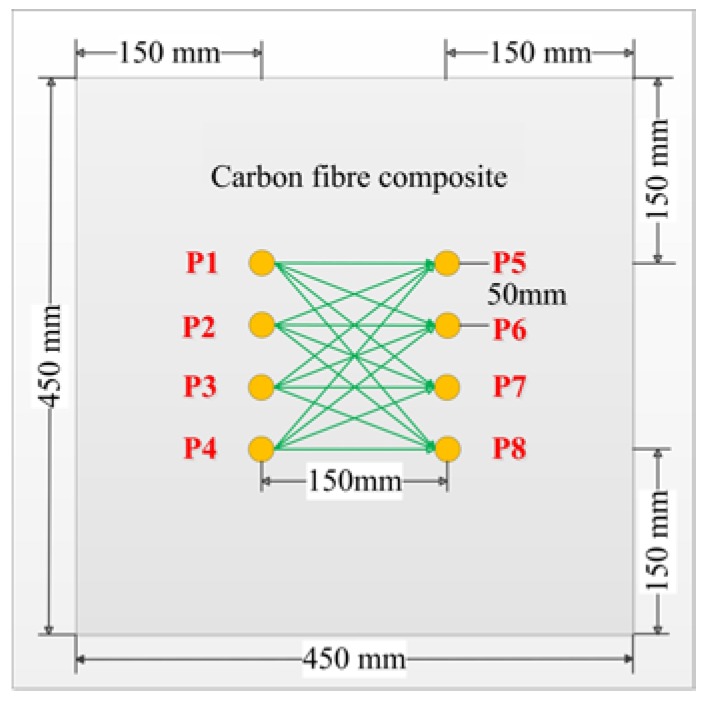
Sensor layout of carbon fiber composite plate.

**Figure 5:**
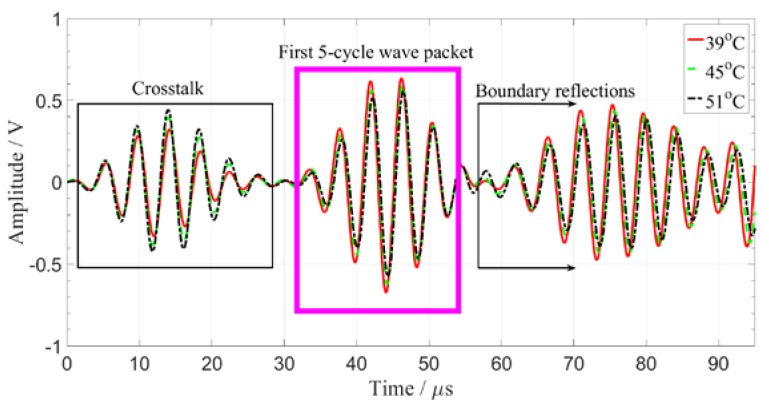
Piezoelectric sensor signals obtained at three temperatures.

Set the oven temperature from 39 to 84 °C with a temperature interval of 3 °C. When the temperature reaches a predetermined value, it is kept for 30 min to reach a steady state. Eight circular piezoelectric sensors (PZT-5, diameter 8 mm, thickness 0.45 mm) were arranged on a carbon fiber composite plate (450 mm × 450 mm × 2 mm) as shown in Figure 4. The PZTs marked by P1–P4 were used as transducers, the other PZTs served as sensors. There are 16 excitation-receiving paths for pitch–catch mode. The distance between the sensor and the board boundary is set to 15 cm in order to separate the direct wave packet from the reflection wave. The typical piezoelectric sensor signals were shown in Figure 5. The excitation frequency is 200 kHz. The first part of the signals is crosstalk, the second part are the direct wave signals, and the third part is mainly the boundary reflection and modal aliasing waves. In order to eliminate interference, the second part of the direct wave signals was taken for processing and analysis. Considering the multimode and dispersion characteristics of ultrasonic guided waves, the proposed method is verified by the following four parts: (1) compensation of guided wave signals with a single frequency; (2) compensation of guided wave signals with different frequencies; (3) compensation of guided wave signals in different paths; (4) multiple compensation of guided wave signals with the same frequency in the same path.

### 4.2. Process of Compensation

**Figure 6:**
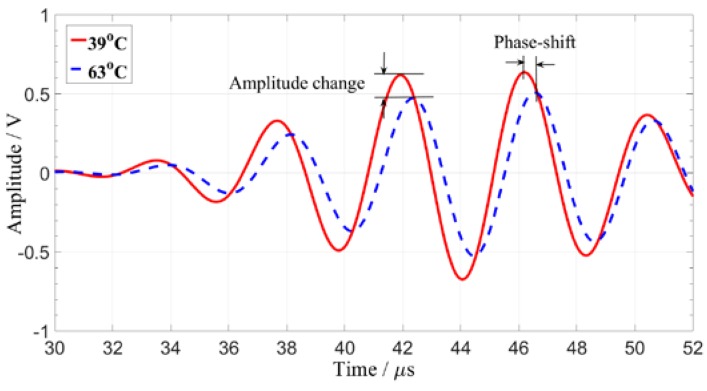
Comparison of guided wave signals at 39 and 63 °C.

Figure 6 shows the ultrasonic guided wave time domain signals obtained at different temperatures. The temperature variation makes a significant change in the amplitude and phase of the guided wave signals. The signal amplitude exhibits uneven variation characteristics. The relationship between phase shift and temperature is determined by the signal feature point (the peak value of the direct wave).

As previously mentioned, f(T) and Coef (T) are obtained first, and then the first compensated signal is obtained by these two relationships. After that, it is compared with the real received signal to calculate the compensation level (abbreviated as CL), and if the value satisfies the given compensation criterion, the compensation is completed. The value of CL is calculated according to Equation (12):

(12) $$CL=20\log\frac{max\left|S_{T}\left(t\right)-S_{c}\left(t\right)\right|}{\max|S_{T}\left(t\right)|}$$

where $S_{T}\left(t\right)$ is the received signal at *T*(°C) and $S_{c}\left(t\right)$ is the compensated signal at *T* (°C). The lower the CL value, the better the compensation effect.

According to the damage test data [17], for a 5 mm × 5 mm damage, the CL value of the residual signal caused by the damage is about −24 dB, and the value of the temperature compensation standard is set to −25 dB.

**Figure 7:**
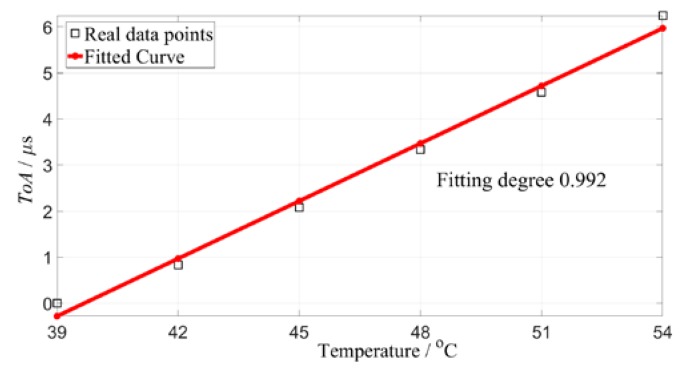
The signal phases at different temperatures.

In the real compensation process, the set of reference signals is first obtained at a given temperature range between 39 and 54 °C. The signal at 39 °C is regarded as the first baseline signal (BS1 for short), and the remaining reference signals are represented as BS2, BS3, ... As shown in Figure 7, as the temperature increases, ToA increases linearly. This linear relationship is used to compensate for phase changes in the measured signals at other temperatures.

**Figure 8:**
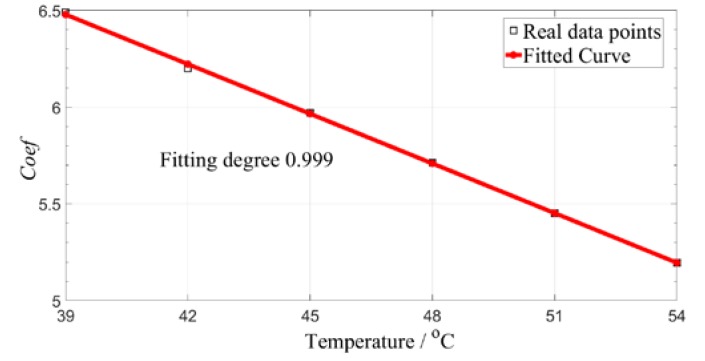
Relationship between temperature and amplitude influence coefficient, Coef.

According to BS1, the Gabor atom matched by the reference is obtained, and the reference signal is matched with the reference matching atom, and a plurality of values is obtained using Equation (10). The relationship between the temperature and Coef is established, as shown in Figure 8. It is easy to find that as the temperature increases, the Coef value decreases linearly, which is consistent with the variation of the amplitude of the guided wave signal under the influence of temperature.

### 4.3. Analysis of Compensation Results

**Figure 9:**
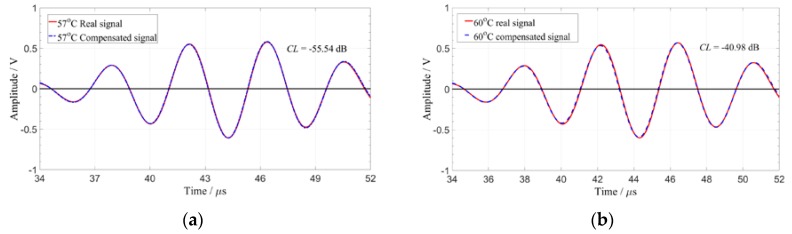

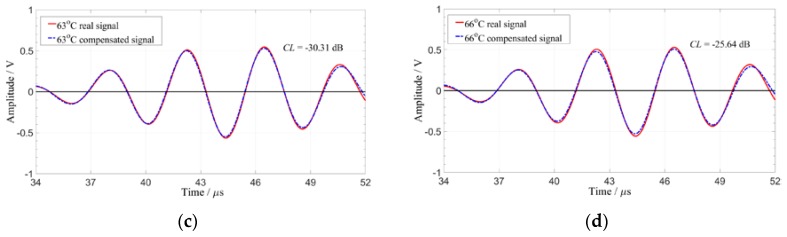
The real signal, compensated signal, and its compensation level (CL) value at four temperatures. (**a**) 57 °C; (**b**) 60 °C; (**c**) 63 °C; (**d**) 66 °C.

According to the obtained amplitude influence coefficient, Coef, and ToA, the compensation signal of the guided wave signal outside the temperature range of the reference signal set is obtained by the interpolation method to eliminate the influence of temperature. Figure 9 and Table 1 show the compensation result of the guided wave signals with the excitation frequency of 200 kHz at four different temperatures for the 4–8 path.

It can be seen from Table 1 that in the case of satisfying the given compensation standard of −25 dB, the signal obtained at 66 °C can be compensated effectively and the temperature interval can reach up to 10 °C.

**Figure 10:**
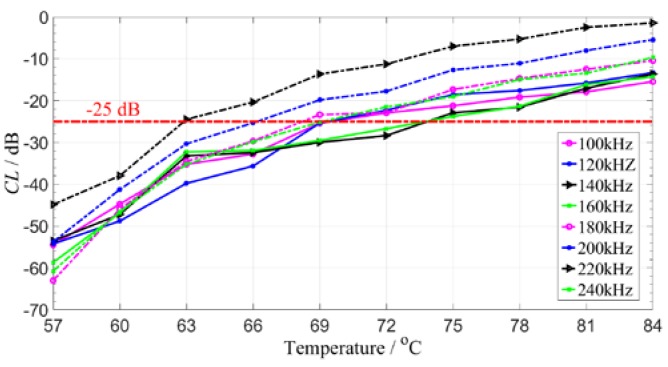
The CL value of guided waves signals with different frequencies at different temperatures.

Using this method, the guided wave signals with different frequencies for the 4–8 path are compensated at different temperatures. The compensation results are shown in Figure 10. It can be found that under the given compensation standard, the method has slightly different compensation effects on the guided signals with different frequencies, mainly due to the dispersion characteristics of the guided waves. Except for the signal with 220 kHz, the ultrasonic guided wave signals can be compensated in a larger temperature range of around 10 °C as shown in Figure 10.

**Figure 11:**
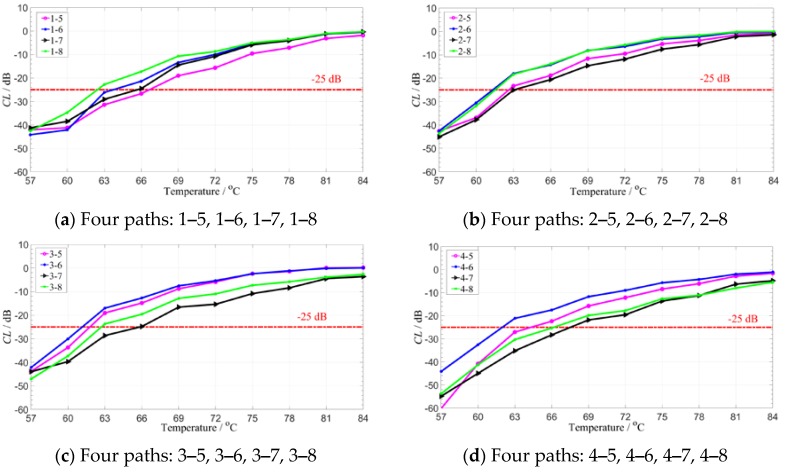
The CL values vary with temperatures at different paths.

Finally, the compensation effects for the signals of different paths are illustrated. The signals with the 180 kHz central frequency at different paths are taken for examples. The signals of 16 paths are compensated and analyzed separately as shown in Figure 11. It can be found that under the given compensation standard, the method has slightly different compensation effects on the guided signals of different paths.

**Figure 12:**
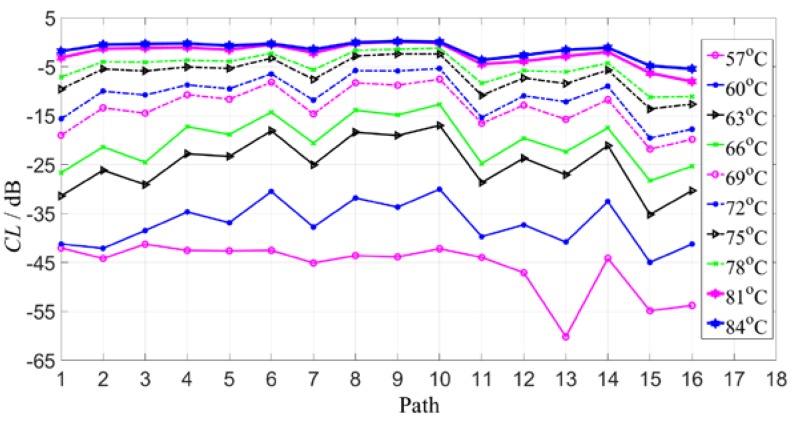
The curves of CL values under different temperature changes with different paths.

It can be concluded from the above results that in the range of 30 °C higher than the reference signal set, as the temperature increases, the CL value tends to be gentle; and the closer the temperature is to the temperature range of the reference signal set, the smaller the compensation level value is. Under the condition of the compensation standard, the temperature compensation variation of different receiving of the same excitation is stable in the range of 61–66 °C. Even if the path with the worst signal reception effect is obtained, the compensation effect can be compensated under the temperature interval of more than 7 °C. Therefore, the real applications should be based on this experience guidance. The different compensation effect of different paths may be related to the difference in piezoelectric sheet performance in practical applications. The 16 paths in Figure 11 are made to change the CL value with the propagation path at the same temperature, as shown in Figure 12.

It can be seen from Figure 12 that except for the partial path (the CL value is small), the CL value after each path compensation is generally stable, so the effect of different paths on the compensation effect can be ignored in practical applications without considering the difference in sensor performance.

As mentioned above, considering the iterative compensation of the residual signal for multiple iterations can further improve the temperature compensation performance of the method. The specific iteration equations are shown in Equations (13) and (14):

(13) $$S_{T}^{1}=Coef_{1}\times g_{b_{1}}+R^{1}S,$$

(14) $$\;S_{T}^{k}=S_{T}^{k-1}+Coef_{k}\times g_{b_{k}}+R^{k}S\;,$$

where k is the number of compensations (*k* = 2, 3, ...), $S_{T}^{k}$ is the signal after the $k^{th}$ compensation at *T* (°C).

**Figure 13:**
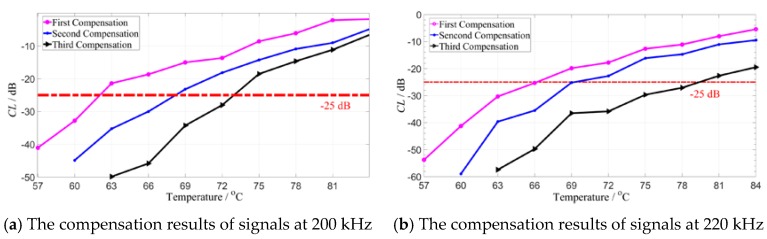
The CL values obtained by different iteration times.

According to above equations, the guided wave signals with the excitation frequency of 200 and 220 kHz in the 1–5 path are compensated for three times in the experimental results. The relationship between the CL value and the temperature after different compensation is shown in Figure 13.

For the first compensation, the compensation temperature interval can reach 10 °C; after the second compensation, the temperature interval reaches 15 °C; and in the third compensation, the temperature interval can reach 25 °C. Even for the guided wave signal with the 220 kHz with the worst compensation for one iteration, the temperature interval after three compensations can reach 18 °C. Therefore, the compensation effect can be further improved by multiple iterations of the residual signals. In this way, a larger temperature compensation interval can be achieved to accommodate more complex environments. Table 2 is a comparison of temperature range and temperature interval for existing temperature compensation methods.

## 5. Experimental Validations of Damage Imaging Based on Temperature Compensation

In this section, the experiments on a specimen with simulated damage are carried out in a temperature-changing environment. The proposed ultrasonic guided wave temperature compensation method based on the reference signal matching is used to eliminate the influence of temperature. The damage diagnosis imaging is performed to illustrate the damage location [33]. The effectiveness of the compensation method is verified by comparing the damage diagnosis imaging obtained without temperature compensation.

**Figure 14:**
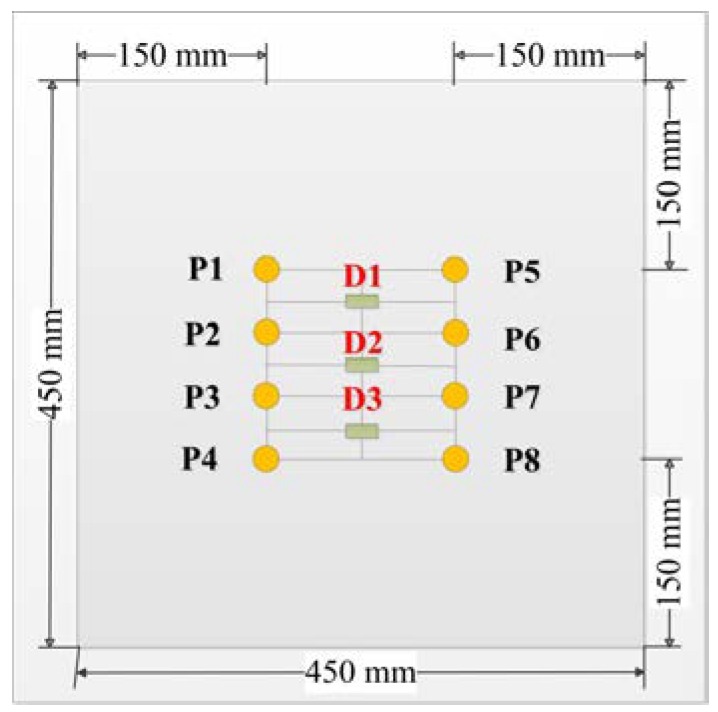
Sensor layout of carbon fiber composite plate.

The presence of damage can cause significant attenuation of the guided wave signal. Therefore, the industrial absorbing adhesive is used to simulate the real damage due to the removable flexibility in the damage diagnosis imaging experiment. Three absorbing adhesives as simulated damages (D1, D2, D3) are bonded to the composite plate mentioned in Section 4.1, respectively, as shown in Figure 14.

**Figure 15:**
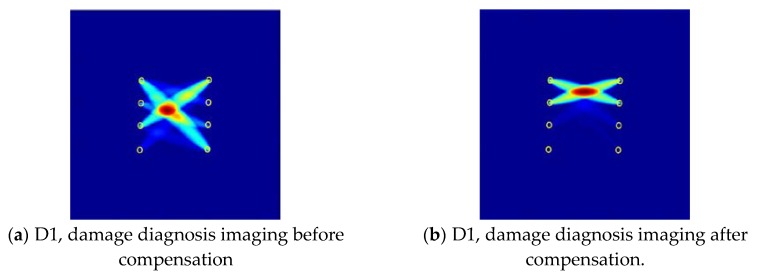

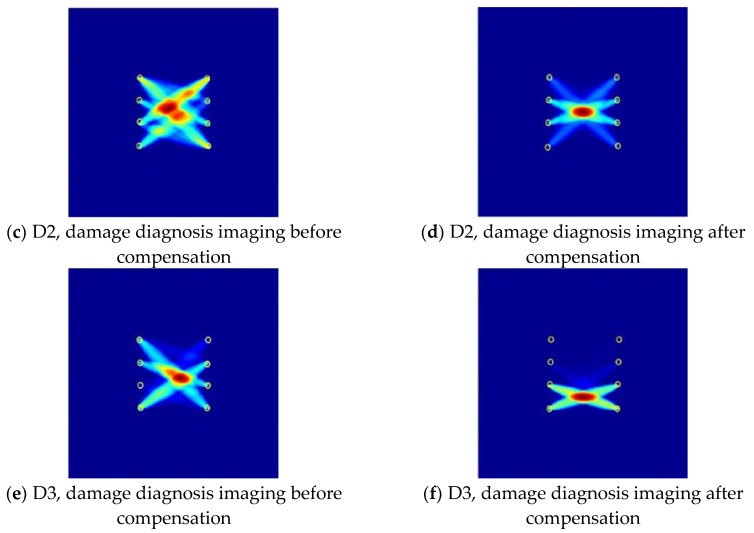
Damage diagnosis imaging of simulated damage before and after temperature compensation in three different positions. (The darker the color in the figure, the greater the probability of damage, and the yellow circle indicates the sensor position.)

Firstly, the reference signal set at 200 kHz is obtained at a given temperature from 39 to 54 °C with 3 °C intervals. The relationship between temperature and ToA is established from the reference set signal. For three cases of simulated damages, the corresponded diagnostic signals are acquired as current measured signals at 66 °C. The relationship established by the reference signal set is used to obtain the compensation signal at the actual temperature. The difference is compared with the measured signal to obtain the residual signal. Finally, the diagnostic imaging algorithm based on probability-weighted distribution [33] is used to obtain the damage diagnosis image after the ultrasonic guided wave temperature compensation, as shown in Figure 15b,d,f.

As shown in Figure 15b,d,f, the identified damage position is basically consistent with the simulated damage position. Figure 15a,c,e is the result of damage imaging directly from the residual signal derived from the reference signal and the measured signal. It can be seen that the position of the directly located damage deviates from the corresponding predefined position due to the influence of temperature. It is indicated that the reference matching guided wave temperature compensation method proposed in this paper can effectively reduce the damage diagnosis error.

## 6. Conclusions

Based on the characteristics of temperature influence on guided wave signals, a temperature compensation method based on reference signal matching is proposed for ultrasonic guided wave signals. The proposed method only needs to select several reference signals in a certain temperature range during the whole compensation process. The temperature compensation range is adjustable, the calculation amount is small, and the application is simple. Experimental results of temperature compensation of guided wave signals show that the compensation can achieve compensation of temperature intervals greater than 7 °C in the path with the worst compensation effect; by performing multiple matching pursuit iterations on the residual signal, the compensated temperature interval can be significantly improved. The signal compensation of a temperature interval greater than 18 °C can be achieved after three iterations. This method has good stability to the change of the guided wave propagation path and frequency.

Future work should be done to verify the effectiveness and reliability of compensation for various environmental factors under different environmental and damage conditions and to verify their effect to detect and locate damage under different temperature conditions.

## Figures and Tables

**Table 1:** The CL value of real signal and compensated signal at four compensation temperatures.

Compensation Temperature	*CL*
57 °C	−55.54
60 °C	−40.98
63 °C	−30.31
66 °C	−25.64

**Table 2:** Comparison of temperature range and temperature interval for existing temperature compensation methods.

Compensation Method	Temperature Range	Maximum Compensation Interval	Reference Signal Interval ^2^
BSS	5–40 °C	5 °C	0.1 °C
OBS	22–32 °C	10 °C	0.1 °C
BSS + OBS	21.5–31.5 °C	10 °C	0.5 °C
Physics-based	30–90 °C	10 °C	4 °C
Reference Matching-based	39–84 °C	25 °C ^1^	3 °C

^1^ Result after multiple iterations of the residual signals; ^2^ The larger the reference signal temperature interval, the smaller the reference data set and the simpler the calculation process. BSS—baseline signal stretch; OBS—optimal baseline subtraction.
